# Molecular identification and genetic diversity among *Photorhabdus* and *Xenorhabdus* isolates

**DOI:** 10.1007/s13205-016-0594-4

**Published:** 2017-04-08

**Authors:** Reda E. A. Moghaieb, Abdelhadi A. Abdelhadi, Hanan A. El-Sadawy, Nesreen A. T. Allam, Baiome Abdelmaguid Baiome, Mohamed H. Soliman

**Affiliations:** 1 Department of Genetics and Genetic Engineering Research Center (GERC), Faculty of Agriculture Cairo University, Giza, 12613 Egypt; 2 College of Biotechnology, University of Modern Science, UAE, 232816 Dubai, United Arab Emirates; 3 Parasitology and Animal Diseases Department, National Research Centre, Elbuhoth St., Dokki, Giza, 12311 Egypt

**Keywords:** *Photorhabdus*, *Xenorhabdus*, 16S rDNA, Molecular markers, Genetic diversity

## Abstract

Five bacterial strains were isolated from the hemocoel of the greater wax moth larvae (*Galleria mellonella*) infected with the entomopathogenic nematodes: *Heterorhabditis bacteriophora* HP88, *Heterorhabditis indicus* RM1 and *Heterorhabditis sp* (S1), *Steinernema abbasi* and *Steinernema* sp. (S II). Strains were identified as *Photorhabdus luminescens* HRM1, *P. luminescens* HS1, *P. luminescens* HP88, *Xenorhabdus indica* and *X. nematophila* ATTC19061 using 16S rDNA sequence analysis. To reveal the genetic diversity among these strains, three molecular markers (RAPD, ISSR and SRAP) were employed. RAPD analysis showed 73.8 and 54.5 polymorphism percentages for the *Photorhabdus* and *Xenorhabdus* strains, respectively. ISSR analysis resulted in 70.1 and 75.2 polymorphism percentages among the *Photorhabdus* and *Xenorhabdus* strains, respectively. The SRAP analysis indicated that 75.6 and 61.2% genetic polymorphism was detected among *Photorhabdus* and Xenorhabdus strains, respectively. The cluster analysis grouped the three *Photorhabdus strains* together in one cluster and the two *Xenorhabdus* strains together in another cluster indicating the phylogenetic relationships among them. The genotype-specific markers detected from the three molecular markers (RAPD, ISSR and SRAP) were sufficient to distinguish between the different bacterial strains tested and can be used in the future IBM program that could be built on the use of these strains.

## Introduction

*Xenorhabdus* spp. and *Photorhabdus* spp. are motile, Gram-negative bacteria belonging to the family Enterobacteriaceae. These bacteria are symbiotically associated with nematodes of the families, *Steinernematidae* and *Heterorhabditidae*, respectively (Forst et al. 1997; Sheets et al. 2011; Ferreira et al. 2014). These symbiotic complexes are highly pathogenic for a wide range of insects and are, therefore, used worldwide as biological agents in crop control (Ehlers 2003; Goodrich-Blair and Clarke 2007; Del Valle et al. 2013).

Many symbiotic bacterial species and isolates are still to be identified or described. Several identification techniques are now available that range from total protein, isozyme profiles to DNA/DNA hybridization and sequence analysis of the 16S rDNA region (Akhurst and Boemare 1990; Boemare and Akhurst 2007; Lee and Stock 2010).

The availability of molecular markers such as 16S rDNA (Christensen et al. 1998), RAPD (Williams et al. 1990; Welsh et al. 1991), ISSR (Zietkiewicz et al. 1994) facilitates the development of phylogenetic relationships, identification and characterization of bacterial species.

The sequence-related amplified polymorphism (SRAP) system was developed by Li and Quiros (2001) to target overlapping coding and non-coding regions of the genome. Depending on the amplification of Open-Reading Frames (ORFs) using the GC-rich exons and the promoter (Li and Quiros 2001), SRAP not only amplifies the interval between genes and their non-coding flanking regions, but also tightly links to actual genes, which would generate a fingerprint of the coding sequences and permit easy isolation of these bands for sequencing (Yu et al. 2008). SRAP is used in genetic map construction, genealogical classification, gene tagging and cloning, population structure, genetic diversity and genetic linkage map of plants (Li et al. 2007; Zheng et al. 2010; Jiang and Liu 2011; Lu et al. 2012; Alghamdi et al. 2012). Furthermore, it was used to study genetic diversity in parasites of human and animal health (Li et al. 2009; Song et al. 2011).

In the present work, five bacterial isolates (three *Photorhabdus* and two *Xenorhabdus*) were identified at the molecular level based on the 16S rDNA region. Furthermore, the genetic polymorphism among the five bacterial isolates was investigated at the molecular levels using RAPD, ISSR and SRAP analyses. The obtained data were used to construct the phylogenetic tree. The genotype-specific markers were also determined.

## Materials and methods

### Bacterial strains and culture

Five bacterial isolates were isolated from the hemocoel of the greater wax moth larvae (*Galleria mellonella*) infected with different entomopathogenic nematode strains (*Steinernema abbasi*, *Steinernema* sp. (S II), *Heterorhabditis bacteriophora* HP88, *Heterorhabditis indicus* RM1 and *Heterorhabditis* sp. (S1)). The bacterial isolates were plated on Nutrient Bromothymol Blue Agar (NBTA) medium (Akhurst 1980). On which Phase I is distinguished from Phase II by its adsorption of bromothymol blue to produce a red core colony overlaid by dark blue and surrounded by a clear zone after 3–4 days of incubation at 25 °C (Wang et al. 2008). The NBTA medium contained 20 g nutrient agar (Difco), 25 mg bromothymol blue (s.d. FiNE_CHEM ltd), and 40 mg tripheyltetrazolium (BDH, England) in 1 L distilled water. The Phase I colony was selectively transferred to 5 mL LB broth (Difco), and incubated at 25 °C for 48 h with gentle agitation (100 rpm).

### 16S rDNA analysis

Genomic DNA was extracted using the nucleic acid extraction kit (Solgent, Korea) following the manufacturer’s instructions. Isolated symbiotic bacteria were identified by nucleotide sequence analysis of 16S ribosomal DNA (rDNA). The universal primer set used was a forward 27F (5′-AGA GTT TGA TCC TGG CTC AG-3′) and a reverse 1492R (5′-GGT TAC CTT GTT ACG ACT T-3′) (Ibrahim et al. 1993). Polymerase chain reaction (PCR) was performed with genomic DNA as a template in a total volume of 50 μl containing 10 mM Tris-HCl (pH 8.3), 50 mM KCl, 2 mM MgCl_2_, 0.2 mM dNTPs, 0.2 pmol of each primer, and *Taq* DNA polymerase (Promega^®^). The amplification was carried out in a DNA thermocycler (MWG BIOTECH Primuse) programmed as follows: (94 °C/4 min) 1, (94 °C/1 min, 58 °C/1 min and 72 °C/1 min) 35, (72 °C/7 min) 1 (Jiang et al. 2006; Shrestha and Lee 2012). The PCR products were eluted from agarose gels using Promega^®^’s Wizard^®^ SV Gel and PCR Clean-Up System according to the manufacturer’s instructions. The purified DNA fragments from each sample were sent for DNA sequencing.

### RAPD analysis

RAPD-PCR was carried out according to the procedure reported by Williams et al. (1990). Eleven primers were used in this study (Table 1). Amplification reaction was carried out in 25 μL volume containing 50 ng of genomic DNA template, 2.0 μM primer (Operon Technology, Inc., Almeda, CA, USA), and 2.0 μM each of dNTPs mix, 2.0 mM MgCl_2_, 1× buffer and 2 units of *Taq* DNA polymerase. The amplification was carried out in a DNA thermocycler (MWG-BIOTECH Primuse) programmed as follows: (94 °C/4 min) 1, (94 °C/1 min, 35 °C/1 min, and 72 °C/1 min) 35, (72 °C/7 min) 1.

**Table 1:** The nucleotide sequences of RAPD, ISSR and SRAP primers used

RAPD primer sequence			
Primer	Sequence (5′–3′)	Primer	Sequence (5′–3′)
OPE-L04	GACTGCACAC	OPE-B04	GGACTGGAGT
OPE-M15	GACCTACCAC	OPE-P09	GTGGTCCGCA
OPE-P15	GGAAGCCAAC	OPE-F12	ACGGTACCAG
OPE-E06	AAGACCCCTC	OPE-K02	GTCTCCGCAA
OPE-D20	ACCCGGTCAC	OPE-Q14	GGACGCTTCA
OPE-B10	CTGCTGGGAC		

ISSR primer sequence			
Primer	Sequence (5′–3′)	Primer	Sequence (5′–3′)
UBC807	(AG)8T	UBC818	(CA)8G
UBC808	(AG)8C	IS3	TTT(TCC)5
UBC809	(AG)8G	IS4	CAT(CA)7T
UBC810	(GA)8T	IS5	ACA(TG)7
UBC811	(GA)8C	IS10	(TCC)5AC
UBC815	(CT)8G	UBC827	(TG)8A

SRAP primer sequences			
Forward primer	Sequence (5′–3′)	Reverse primer	Sequence (5′–3′)
Me1	TGAGTCCAAACCGGATA	Em2	GACTGCGTACGAATTTGC
Me2	TGAGTCCAAACCGGAGC	Em3	GACTGCGTACGAATTGAC
Me5	TGAGTCCAAACCGGAAG	Em4	GACTGCGTACGAATTTGA
Me6	TGAGTCCAAACCGGTAG	Em5	GACTGCGTACGAATTAAC
Me10	TGAGTCCAAACCGGGAC	Em6	GACTGCGTACGAATTGCA

### ISSR analysis

ISSR amplification was performed according to Kafkas et al. (2006) using 12 primers (Table 1). Amplification reaction was carried out in 30 μL volume containing 50 ng of genomic DNA template, 2.0 μM primers, 2.0 μM each of dNTPs mix, 2.0 mM MgCl_2_, 1× buffer and 2 units of *Taq* DNA polymerase. The amplification was carried out in a DNA thermocycler (MWG-BIOTECH Primuse) programmed as follows: (94 °C/4 min) 1, (94 °C/1 min, 40–60 °C/1 min, 72 °C/1 min) 40, and (70 °C/5 min) 1.

### SRAP analysis

Twenty-five SRAP combinations of five forward and five reverse primers were used (Table 1). The polymerase chain reaction was carried out according to Li and Quiros (2001) and the modification of Baloch et al. (2010). Amplification reaction was carried out in 30 μL volume containing 50 ng of genomic DNA template, 2.0 μM forward primer, 2.0 μM reverse primer, and 2.0 μM each of dNTPs mix, 2.0 mM MgCl_2_, 1× buffer and 2 units of *Taq* DNA polymerase. The amplification was carried out in a DNA thermocycler (MWG-BIOTECH Primuse) programmed as follows: (94 °C/4 min) 1, (94 °C/1 min′, 35 °C/1 min, 72 °C/1 min) 5, (94 °C/1 min, 50 °C/1 min, 72 °C/1 min) 35, and (70 °C/5 min) 1.

### Data analysis

All the genotypes were scored for the presence and absence of the RAPD, ISSR and SRAP bands. And the data were entered into a binary matrix as discrete variables, 1 for presence and 0 for absence of the character and this data matrix was subjected to further analysis.

The Excel file containing the binary data was imported into NT Edit of NTSYS-pc 2.02J. The 0/1 matrix was used to calculate similarity as DICE coefficient using SIMQUAL subroutine in SIMILARITY routine. The resultant similarity matrix was employed to construct dendrogram using sequential agglomerative hierarchical nesting (SAHN) based on the unweighted pair group method with arithmetic means (UPGMA) to infer genetic relationships and phylogeny (Sneath and Sokal 1973).

## Results and discussion

**Fig. 1:**
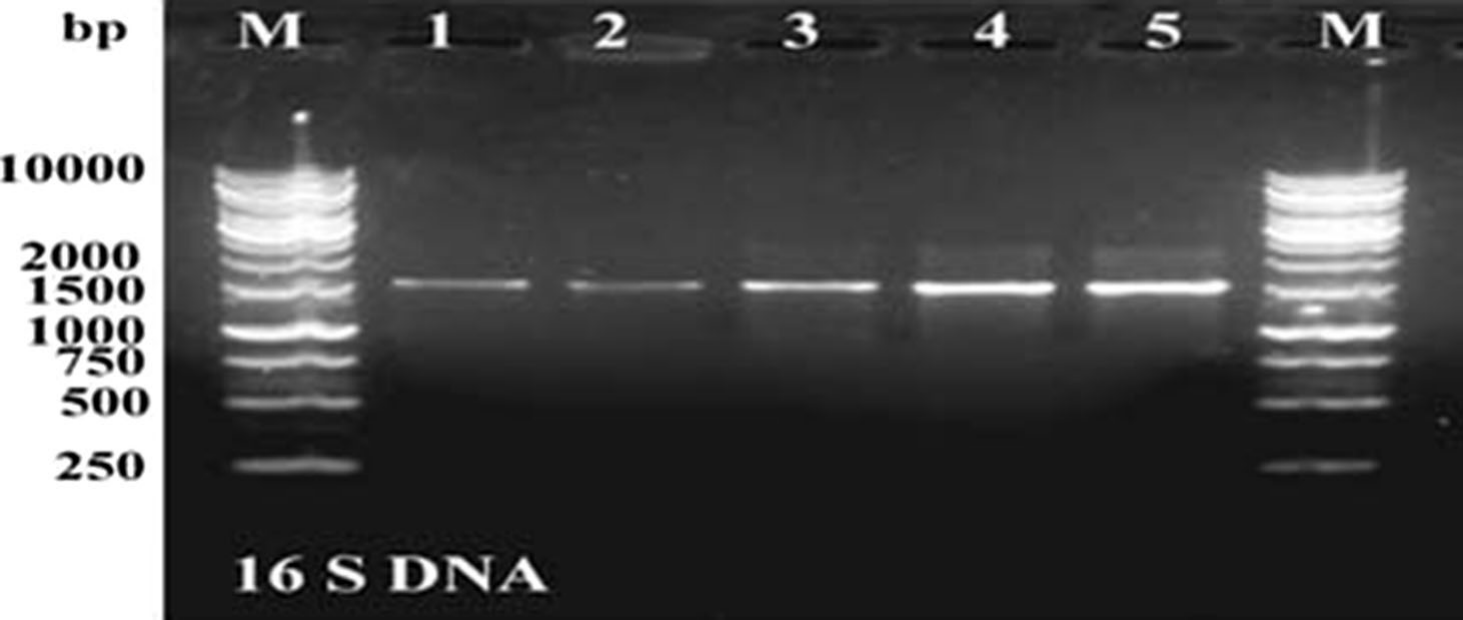
PCR profile of the 16S rDNA for the five bacterial isolates. *M* the 1 kb ladder. *Lanes 1*–*5* the bacterial strain (*lane 1*: *p.HP88*, *lane 2*: *p.RM1*, *lane 3*: *p.S1*, *lane 4*: *x. Ab*, *lane 5*: *x.s2*)

In the present study, three symbiotic bacterial strains isolated from *Heterorhabditis* family and two from *Steinernema* family were identified using 16S rDNA sequence analysis. The data indicated that 1530 bp fragment was amplified; these bands were eluted and sequenced (Fig. 1). The complete 16S rDNA gene sequences of all strains (1530 nucleotides) were aligned to the homologous sequences of *Photorhabdus* and *Xenorhabdus* using the BLASTN program in the NCBI (Altschul et al. 1990). The data indicate that the three symbiotic bacterial strains that have been isolated from *Heterorhabditis* family were highly similar to *Photorhabdus*. The identified bacterial strains were registered on the GeneBank under the accession number KC237382 to *P. luminescens* HRM1 and KC237383 for the *P. luminescens* HS1, while the third sequence was identical with *P. luminescens* HP88. The data of the two symbiotic bacteria which have been isolated from Stinernema family indicate that one of them was identical to *X. indica* and the second sequence was similar to *X. nematophila* ATTC19061 16S rDNA gene sequence with the score of 96%.

The five bacterial strains identified were characterized at the molecular levels using three molecular markers (RAPD, ISSR and SRAP) to determine the genetic polymorphism among them and also to determine the genotype-specific markers for each strain.

**Fig. 2:**
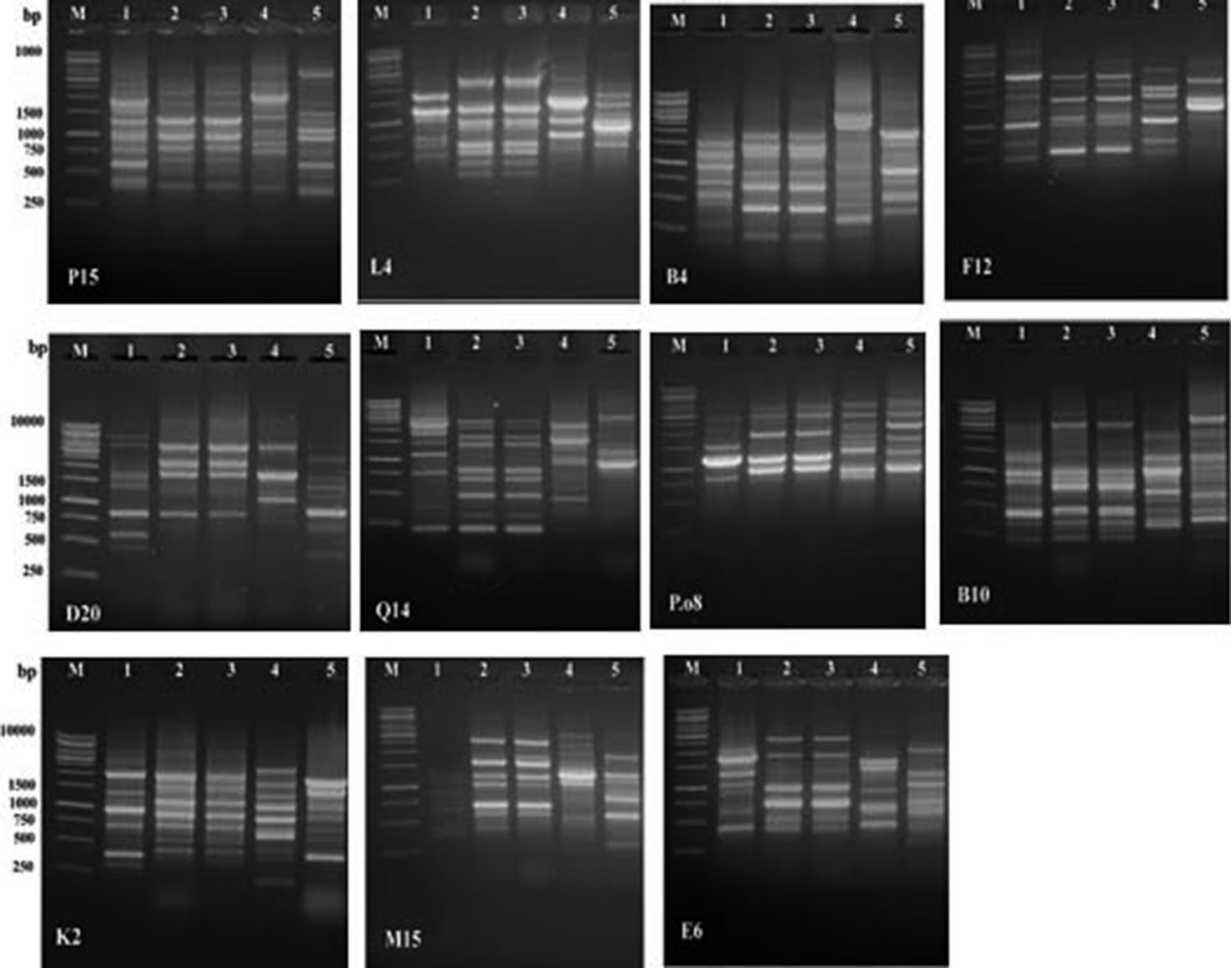
RAPD profiles of the five bacterial strains. *Lanes 1–3* are *Photorhabdus* and *lanes 4–5* are *Xenorhabdus* strains. *M* DNA ladder

Eleven random RAPD primers were used to detect the genetic polymorphism among these five bacterial strains. All the primers tested resulted in clear bands (Fig. 2). The three *Photorhabdus* strains generated 126 bands: 93 bands out of them were polymorphic and can be considered as useful bands for the three bacterial isolates with 73.8 polymorphism percentages. The *Xenorhabdus* strains showed 123 total bands and 76 bands out of the total bands were polymorphic with 54.5% polymorphism (Table 2). The RAPD genotype-specific bands as presented in Table 3 indicate that the three *Photorhabdus* strains showed 41 genotype-specific markers that represent 44% from the polymorphic band detected and 32.5% from the total band numbers (Tables 2, 3). The highest genotype-specific number was recorded for the bacterial strain *P. luminescens* HP88 (37 markers) followed by *P. luminescens* HS1 (2 markers) and *P. luminescens* RM1 (2 markers). *Xenorhabdus* strains showed 76 specific markers: 39 markers of them are specific to *X. nematophila* S2 and 37 for *X. indica* AB. The genotype-specific markers obtained for *Xenorhabdus* strains represent 61.7% of the total band number and 100% of the polymorphic bands (Tables 2, 3).

**Table 2:** Genetic polymorphism among three *Photorhabdus* and two *Xenorhabdus* strains as revealed by RAPD, ISSR and SRAP analyses

	*Photorhabdus* strains				*Xenorhabdus* strains		
	RAPD	ISSR	SERAP		RAPD	ISSR	SERAP
Total number of bands	126	95	230	Total number of bands	123	113	191
Polymorphic bands	93	67	174	Polymorphic bands	76	85	117
Polymorphism %	73.8	70.1	75.6	Polymorphism %	54.5	75.2	61.2

**Table 3:** RAPD genotype-specific markers

Strain	Marker	Total
*P. luminescens* HP88	L4(1750, 1200, 700), M15(400), P15(1700, 800, 500, 350, 200), E6(1600, 1200, 900, 800, 600, 400), D20(1500, 600, 400), B4(900, 500, 350), P.09(1500), F12(4000, 2400, 700, 500), Q14(4000, 400), K2(2500, 1200, 750, 250), B10(2000, 1400, 1200, 600, 450)	37
*P. luminescens RM1*	K2(100), B10(3000)	2
*P. luminescens HS1*	P15(2000), P.09(5000)	2
Total		41
*X. indica AB*	M15(1550, 600, 500), P15(2000, 1700, 1500, 1400, 1200,950, 700, 500), E6(1200, 1000, 250), D20(3000, 1750, 1200), B4(3000, 2200, 400, 250), P.09(2000, 1500, 800), F12(3000, 2400, 1700, 700, 500), Q14(4000, 2000, 1750, 600, 450), B10(800, 700, 400)	37
*X. nematophila S2*	L4(1750, 1200, 900, 600,450, 350, 200), M15(2000, 100, 700, 350), P15(3000, 1000, 900, 750, 350, 250, 100), E6(2000, 1600, 900, 500, 400, 350, 150), D20(2000, 1500, 800, 700, 400), B4(900, 700, 650, 500, 450), F12 (3500), Q14(3000), B10(2500, 750)	39
Total		76

**Fig. 3:**
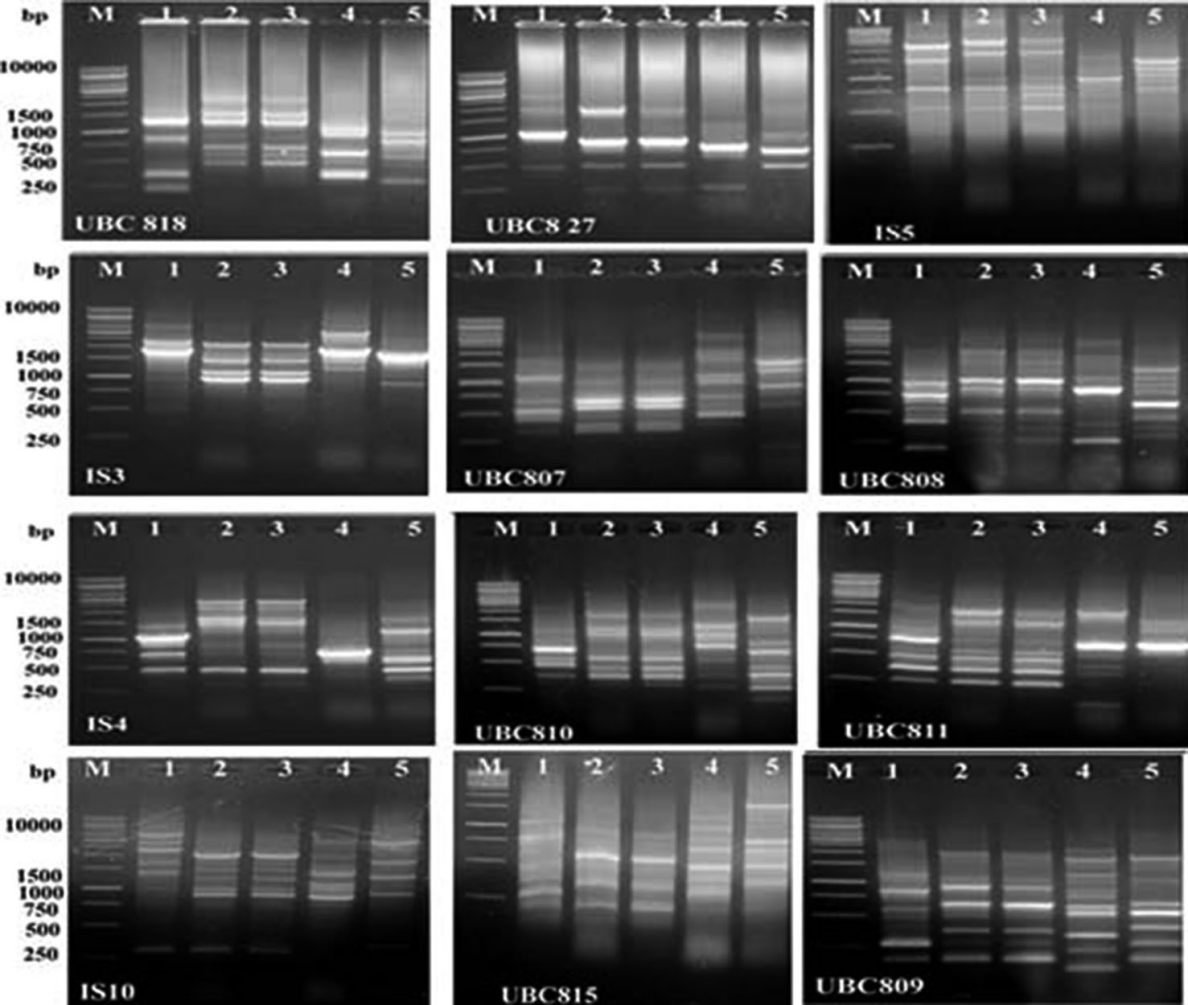
ISSR profiles of the five bacterial strains. *Lanes 1–3* are *Photorhabdus* and *lanes 4–5* are *Xenorhabdus* strains. *M* DNA ladder

The presence of simple sequence repeats (SSR) in prokaryotes is well documented (Gur-Arie et al. 2000), and some SSRs show extensive length polymorphisms (Yang et al. 2003; Sreenu et al. 2006). Successful use of PCR-based SSR amplification followed by amplicon size determination to analyze the spread of microbial pathogens has been reported for *Haemophilus influenzae* and *Candida albicans* (Bretagne et al. 1997; van Belkum et al. 1997). Furthermore, the utility of inter-simple sequence repeat-PCR (ISSR-PCR) assay in the characterization and elucidation of the phylogenetic relationship between the pathogenic and nonpathogenic isolates of *Vibrio cholerae* was demonstrated (Kumar et al. 2007). They proposed that ISSR-PCR is an efficient tool in phylogenetic classification of prokaryotic genomes in general and diagnostic genotyping of microbial pathogens in particular. A DNA sample representing the five bacterial strains was subjected to PCR analysis using twelve ISSR primers. The primers used show stable and repeatable banding pattern for the strain tested (Fig. 3). Data presented in Table 2 indicate that 95 ISSR bands were generated among the three *Photorhabdus* strains; 28 bands out of total band numbers were monomorphic bands representing a common band in the genus *Photorhabdus* and 67 bands which are polymorphic can be considered as useful bands for the three bacterial isolates with 70.1 polymorphism percentages. The primers showed different polymorphism percentages that ranged from 33% for the primer IS3 to 100% for UBC815. The *Xenorhabdus* strains showed 113 total bands; 85 bands out of the total bands were polymorphic with 75.2% polymorphism (Table 2).

The ISSR genotype-specific bands were determined. Data presented in Table 4 show that ISSR marker can distinguish between the different bacterial strains. The three *Photorhabdus* strains showed 34 genotype-specific ISSR markers that represent 50.7% from the polymorphic band detected and 75.2% from the total band numbers (Table 4). The highest genotype-specific number was recorded for the bacterial strain *P. luminescens* HP88 (21 markers) followed by *P. luminescens* RM1 (9 markers) and *P. luminescens* HS1 (4 markers). *Xenorhabdus* strains showed 85 specific markers: 45 markers of them are specific to *X. nematophila* S2 and 40 for *X. indica* AB. The genotype-specific markers obtained for *Xenorhabdus* strains represent 75.2% of the total band numbers and 100% of the polymorphic bands (Table 4).

**Table 4:** ISSR genotype-specific bands among *Photorhabdus* and *Xenorhabdus* strains

Strain	Marker	Total
*P. luminescens* HP88	IS3(1200, 500), IS4(900, 750, 400), IS5(1200), UBC807(1000, 400), UBC808(900, 750, 650, 600, 400, 200), UBC827(3000, 1050), UBC809(800, 600), IS10(700), UBC810(2500, 2200)	21
*P. luminescens RM1*	UBC809(1600), IS10(1400, 1100, 1000), UBC810(2000, 1000, 800, 750, 100),	9
*P. luminescens HS1*	IS5(300),UBC827(1250), IS10(260), UBC810(1250)	4
**Total**		34
*X. indica AB*	IS3(3500, 2200, 1200), IS5(400, 300), UBC807(2500, 2000, 1800, 1000, 900, 530, 400), UBC808(360, 250), UBC827(670, 220), UBC809(2000, 1500, 650, 520, 500, 230, 150), IS10(2000, 1100, 900, 700), UBC810(1500, 1250, 1000, 800, 750,100), UBC811(1500, 1350, 1550, 1500, 350, 300, 100)	40
*X. nematophila S2*	IS3(4000, 1800, 1000, 900, 600), IS4(3000, 300, 200, 150), IS5(3500, 1100, 780), UBC815(1900, 750, 350), UBC807(1400, 1200, 800, 500, 150), UBC808(1200, 900, 750,650, 600, 500, 450,350,300), UBC827(1050, 750, 490), UBC809(350, 250, 200), IS10(1400, 600, 550, 300, 250, 150), UBC810(2000, 1550, 350), UBC811(1350, 250)	45
**Total**		85

Li and Quiros (2001) reported that the SRAP forward primer preferentially amplified exonic regions and the reverse primer preferentially amplified intronic regions. Therefore, the SRAP technique could detect polymorphisms arising from variations in the length of introns, promoters, and spacers, among both genotypes and species when different forward and reverse primers are randomly combined (Li et al. 2014).

**Fig. 4:**
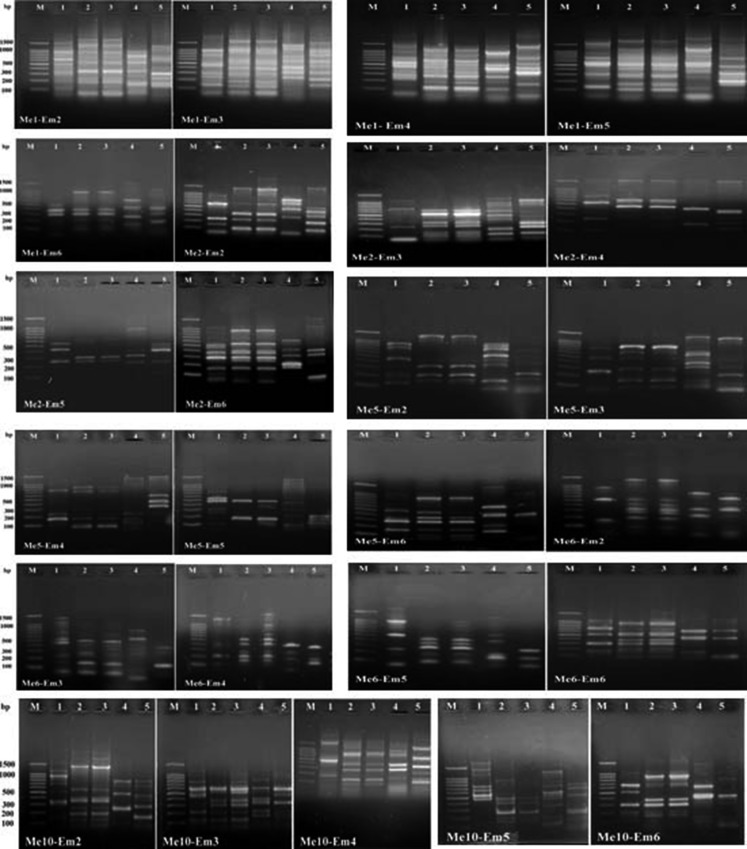
SRAP profiles of the five bacterial strains. *Lanes 1–3* are *Photorhabdus* and *lanes 4–5* are *Xenorhabdus* strains. *M* DNA ladder

In the present study, twenty-five SRAP combinations of five forward and five reverse primers were used for fingerprinting the five bacterial strains. All the SRAP primer combinations resulted in a scorable and reproducible bands. The data presented in Fig. 4 illustrate the SRAP banding pattern of the five bacterial strains.

Data presented in Table 2 indicated that 230 bands were generated among the three *Photorhabdus* strains: 174 bands out of the total band numbers were polymorphic and can be considered as useful bands for the three bacterial isolates with 75.6 polymorphism percentages. The primer combinations showed different polymorphism percentages. The *Xenorhabdus* strains showed 191 total bands: 117 bands out of the total bands were polymorphic with 61.2% polymorphism (Table 2).

The SRAP genotype-specific bands were determined. Data presented in Table 5 indicate the possibility of identifying the five bacterial strains based on the SRAP-specific markers. The three *Photorhabdus* strains showed 69 genotype-specific markers that represent 39.6% from the polymorphic band detected and 30% from the total band numbers (Tables 2, 5). The highest genotype-specific number was recorded for the bacterial strain *P. luminescens* HP88 (56 markers) followed by *P. luminescens* HS1 (9 markers) and *P. luminescens* RM1 (4 markers). *Xenorhabdus* strains showed 117 specific markers: 45 markers of them are specific to *X. nematophila* S2 and 72 for *X. indica* AB. The genotype-specific markers obtained for *Xenorhabdus* strains represent 61.2% of the total band number and 100% of the polymorphic bands (Tables 2, 5).

**Table 5:** SRAP genotype-specific markers

Strain	Marker	Total
*P. luminescens* HP88	Me1* Em2(750, 700, 500,280), Me1* Em3(1600, 100), Me1* Em4(1500, 1200, 700, 500), Me1* Em5(120), Me1* Em6(420), Me2* Em2(150,50), Me2* Em3(1200, 700), Me2* Em4(800, 600 200), Me2* Em5(650, 500), Me2* Em6(1100, 500, 100), Me5* Em2(800, 600, 100), Me5* Em3(600, 50), Me5* Em4(600), Me5 *Em5(800, 600,300, 250, 100), Me5* Em6(1000, 500), Me6* Em3 (1300, 1100, 700, 200), Me6* Em4(1200), Me6* Em5(800, 600), Me6* Em6(1000), Me10* Em2(1100, 900, 800, 150), Me10* Em3(450), Me10* Em5(1000, 900, 850,600, 500), Me10* Em6(500)	56
*P. luminescens RM1*	Me2* Em5(700), Me10* Em5(800, 150, 100)	4
*P. luminescens HS1*	Me1* Em5(900, 700, 150), Me5* Em3(350), Me5 *Em5(350), Me6* Em4(1600, 600), Me10* Em3(1000), Me10* Em4(500)	9
Total		69
*X. indica AB*	Me1* Em2(750, 650, 300, 100), Me1* Em3(1300, 1200, 700, 600, 180, 100), Me1* Em4(800, 300), Me1* Em5(120), Me1* Em6(1600, 600, 260, 100), Me2* Em2(800, 600,500 300, 50), Me2* Em3(1200, 600,150), Me2* Em5(900, 300), Me2* Em6(600, 300, 200), Me5* Em2(800, 700, 600, 500, 400, 300, 150), Me5* Em3(500, 400, 350, 300), Me5* Em4(1500, 200, 150), Me5 *Em5(1200, 1000, 900, 850, 800, 700, 600, 500, 350, 300, 1200), Me5* Em6(700, 200), Me6* Em3(900, 700), Me6* Em5(1200, 600), Me6* Em6(180), Me10* Em3(800, 350, 200), Me10* Em4(800,300), Me10* Em5(400, 300), Me10* Em6(1400, 500, 200)	72
*X. nematophila S2*	Me1* Em2(1000, 700, 450, 280), Me1* Em3(80), Me1* Em4(1500, 700, 350), Me1* Em5(300, 250), Me1* Em6(180), Me2* Em2(400, 350, 150, 100), Me2* Em3(100, 300, 220), Me2* Em4(400, 150), Me2* Em5(650, 550), Me2* Em6(350, 100), Me5* Em2(200), Me5* Em4(1600, 700, 600, 500, 250(,Me5 *Em5(150, 100), Me5* Em6(550), Me6* Em3(150), Me6* Em6(1000), Me10* Em2(450, 150), Me10* Em3(1000, 450), Me10* Em4(500, 250), Me10* Em5(800, 700, 200, 150)	45
Total		117

**Fig. 5:**
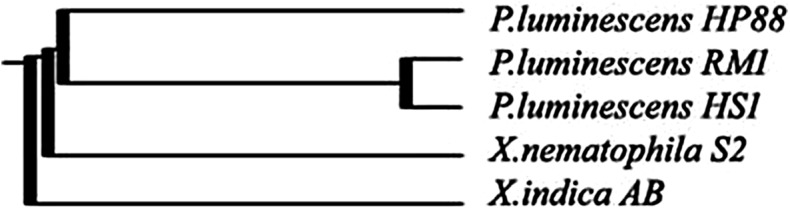
Phylogenetic tree based on the data obtained from RAPD, ISSR and SRAP analyses

The data obtained from the three molecular markers used were pooled together and were used to construct the phylogenetic tree that shows the genetic relationship among the five bacterial strains. The cluster analysis is divided into three clusters: the first cluster has *X. indica* AB, the second cluster has the strain *X. nematophila* S1 while the third cluster is divided into two sub-clusters. The first *P. luminescens* HRM1 and *P. luminescens* HS1 are close together and the other sub-cluster has *P. luminescens* HP88 (Fig. 5). From the cluster analysis, it is clear that the three molecular markers used can distinguish between the five bacterial strains. The *Photorhabdus* strain was grouped together at the same cluster (Fig. 5).

## Conclusion

Based on the data obtained from the present study, the three molecular markers (RAPD, ISSR and SRAP) in addition to the 16S rDNA could be efficiently used to identify and characterize the five bacterial strains. The genotype-specific markers were sufficient to distinguish between the different bacterial strains and can be used in the future IBM program that could be built on the use of these strains.
